# Transcriptomic response to GABA-producing *Lactobacillus plantarum* CGMCC 1.2437^T^ induced by L-MSG

**DOI:** 10.1371/journal.pone.0199021

**Published:** 2018-06-12

**Authors:** Kejin Zhuang, Yujun Jiang, Xiaohan Feng, Li Li, Fangfang Dang, Wei Zhang, Chaoxin Man

**Affiliations:** Key Laboratory of Dairy Science, Ministry of Education, College of Food Science and Technology, Northeast Agricultural University, Harbin, China; Maharshi Dayanand University, INDIA

## Abstract

Gamma-aminobutyric acid (GABA) is an inhibitory neurotransmitter found in the central nervous system of mammals. A range of bacterial species can synthesize GABA, including *Lactobacillus plantarum* of which L-monosodium glutamate (L-MSG) is an inducer of its production. In order to synthesize GABA in high concentrations, L-MSG was utilized as the single inducing factor, a chemically defined medium (CDM) was used as the fermentation substrate, with *L*. *plantarum* CGMCC 1.2437^T^ cultured in medium supplemented with or without L-MSG. High-throughput transcriptome sequencing was used to explore the differential genes expression of bacterial cells at 36 h of fermentation, where the GABA concentration of CDM with L-MSG reached the peak value and was 7.7 times higher than that of medium without L-MSG at the same timepoint. A total of 87 genes showed significant differential expression induced by L-MSG: of these, 69 were up-regulated genes and 18 were down-regulated. The up-regulated genes were assigned to biological processes and molecular function, while the down-regulated genes covered biological process, cellular process and molecular function. Interrogation of results using Gene Ontology (GO) and Kyoto Encyclopedia of Genes and Genomes (KEGG) analyses, indicated carbohydrate metabolism, fatty acid synthesis and amino acid metabolism were closely associated with GABA synthesis induced by L-MSG. This study provides insights into *L*. *plantarum*-mediated GABA fermentation at the molecular level and will provide a new approach for further studies related to GABA production by the other Lactic acid bacteria.

## Introduction

Gamma-aminobutyric acid (GABA) is a non-protein amino acid with several well-characterized physiological functions [1]. GABA can relieve hypertension and anxiety disorders as a major inhibitory neurotransmitter in the mammalian central nervous system [2,3], and can positively affect the recovery of alcohol-related symptoms [4]. Besides, it is also a strong secretagogue of insulin from pancreatic beta cells [5], which can mitigate diabetic vascular complications effectively [6]. Due to diverse beneficial bioactivities of health-related GABA, many researchers focused on how to synthesize high levels of GABA [7]. LAB is a developing field in food, yoghourt, beverage, and dairy food, and LAB have huge potential for GABA production in the food and pharmaceutical industry [8,9]. *Lactobacillus plantarum* [10,11], *L*. *brevis* [10,12], *Lactococcus lactis* [10], *L*. *paracasei* [13], *L*. *delbrueckii* subsp.*bulgaricus* [10], and *Streptococcus salivarius* are generally recognized as safe sources to produce GABA in an eco-friendly way.

Generally, GABA can be easily enriched from cultures, but the capacity of GABA production varies depending on individual strains of LAB, and is also significantly influenced by fermentation conditions, such as temperature, pH, and medium compositions (carbon sources, nitrogen sources and other necessary components required for its accumulation) [1,14]. For most GABA-producing LAB, glutamic acid or glutamate can be irreversibly α-decarboxylated into GABA and play a vital role when used as the substrates of the enzymatic reaction in GABA synthesis [13]. However, in some cases, the superfluous addition of glutamic acid has the ability to inhibit cell growth and to decrease GABA accumulation [15]. Glutamic acid decarboxylase (GAD), localized in the cytoplasm of most GABA-producing LAB, has been reported to be the sole enzyme of glutamate decarboxylation to GABA [16]. So far, to increase the yield of GABA, GAD-encoding genes which are homologously or heterologously expressed in recombinants were detected and cloned from many strains of *Lactobacillus* and *Lactococcus* [17–20]. These researches provide an initial strategy for increasing the yield of GABA at gene level. Moreover, in the downstream of GABA decarboxylation, two other enzymes, GABA/α-ketoglutarate aminotransferase (GABA-AT) and succinic semialdehyde dehydrogenase (SSDH) were also discovered in the constitute GABA shunt pathways together with GAD in some bacteria [21,22]. However, the molecular mechanisms of GABA metabolism (e.g. GABA shunt) are not extensively or comprehensively investigated in bacteria, especially in LAB. Therefore, finding a way to increase the yield of GABA produced by LAB, identification of related genes which effect the synthesis of GABA are vital [23–25].

In the omics era, the study of metabolic pathways and their regulation in microorganism is shifting from enzyme characterization and individual functional genes characterization to whole genome and transcriptome analysis [26–31]. Transcriptome analysis quantifies the changing expression levels of each transcript in cells under different conditions [32], so it is a more effective way to explore complex global metabolism in microorganisms. Presently, various technologies have been developed to deduce and quantify the transcriptome, including hybridization-based or sequence-based approaches [33]. Hybridization-based approaches (e.g. DNA microarrays) are widely applied because of high throughput and relative lower cost. However, high background levels owing to cross-hybridization [34], few dynamic ranges of detection [32] and low reproducibility in different experiments [35] largely limit applications of transcriptome interpretation. In contrast to microarray methods, sequencing technologies of RNA (such as Solexa/Illumina RNA-seq) have been developed to offset these disadvantages. These sequence-based technologies can directly identify cDNA sequences at a large scale and provide a digital precise gene expression measurement [32]. RNA-seq refers to whole transcriptome shotgun sequencing by which mRNA and cDNA are mechanically fragmented and overlapping short fragments covering the entire transcriptome are produced [36]. Moreover, large scale biological information is analyzed to facilitate gene expression quantification, and gene function annotation which provides comprehensive insights into the transcriptome and its regulation. Besides that, RNA-seq can unravel transcriptome complexity and predict structures of transcripts and alternative splicings [36]. RNA-seq has been successfully applied in examining differentially expressed genes (DEGs) and predicting relevant metabolic pathways in eukaryotes and prokaryotes under various stresses and specific conditions [36–38]. Transcriptomic analysis has been used to detect glutamate-induced metabolic changes in *Lactococcus* [39].However to date transcriptomic analysis of glutamate-induced metabolism in *Lactobacillus* has not been reported.

In this study, we developed a fermentation system to produce high quantities of GABA using *L*. *plantarum* CGMCC 1.2437^T^. RNA-Seq was applied to explore the transcriptome changes which are closely related to the production of GABA induced by L-monosodium glutamate (L-MSG). Through this analysis genes which were intimately associated with GABA yield were identified, and metabolic pathways were predicted. These results provide new insights to direct further researches.

## Materials and methods

### Bacterial strain, medium and culture conditions

*L*. *plantarum* CGMCC 1.2437^T^ was purchased from Chinese General Microbiological Culture Collection Center. The strain, maintained in an ampoule tube, was recovered by Lactobacilli MRS agar (Difco, USA) containing 0.5% (*w/v*) CaCO_3_. 16S rDNA and Intergenic spacer (IGS) sequencing were used to confirm the identify of the strain. The bacteria cultures were propagated in two steps: a single colony with a clear zone on plates was inoculated in 25-ml seed medium (Lactobacilli MRS broth, Difco, USA) and then grown statically overnight at 30°C under an anaerobic environment (bio-bag, AnaeroPack-Anaero, Mitsubishi Gas Chemical Company, Inc., Japan). Two ml seed was then incubated in 100 ml MRS broth to 10^8^ cfu ml^-1^ for subsequent fermentation. The pH of seed medium was adjusted to 5.8 before inoculation. The propagated strain was preserved in the MRS medium supplemented with 40% glycerol at -20°C.

### Fermentation for GABA accumulation

Before the formal experiment, we explored optimal fermented conditions for maximum accumulation of GABA by single-factor experiments, with optimal conditions comprising: : concentration of L-MSG (99% purity, Sigma, USA), 100mM; concentration of phosphopyridoxal (PLP, Beijing Biotopped Science & Technology Co. Ltd, China), 100 μM; concentration of CaCl_2_, 10mM; pH of initial fermentation, 5.5; fermented temperature, 37°C (data not shown). Among the five parameters mentioned above, L-MSG was selected as the key limiting factor in comparative fermentation for transcriptomic analysis because of it modulated GABA production significantly. The comparative fermentation was performed in a 2 L flask with chemically defined medium (CDM) according to the reference method [31]. Compared with general fermentation (control group without L-MSG), 100 mM L-MSG was supplemented into a comparative fermentation (experimental group with L-MSG); all other fermention conditions were consistent with the single-factor experiments mentioned above. LP_CTL1 and LP_CTL2 were two replicates of the control group; LP_MSG1 and LP_MSG2 were two replicates of the experimental group. The biomass was determined by spectrophotometric measurement at 600 nm (Beckman DU800, 1 absorbance unit = 0.3 g l^-1^). The change in pH value and GABA production were monitored during the whole fermentation [30].

### GABA measurement

Accumulated GABA in the culture medium was measured as follows. First, the culture broth was separated from cells by centrifugation (10000 × *g* for 10 min at 4°C), and the supernatant was filtered using a 0.2 μm membrane filter (Pall Acrodisc^®^Syringe Filters, USA). Then, GABA concentrations of the supernatant were determined by pre-column derivatization with *o*-phthaldialdehyde reagent (97% purity, HPLC grade, Sigma, USA) [39]. Separations were performed by HPLC (Agilent, 1100 Series, USA) in conjunction with a Hypersil ODS2 C_18_ column (Dalian Elite Analytical Instruments Co. Ltd., China) at 30°C, and the wavelength of the UV detector was 338 nm. The mobile phase was a mixture of sodium acetate (0.05 M, 500 mL), methanol (490 mL, chromatographic grade, Tianjin Guangfu Fine Chemical Co., Ltd., China) and tetrahydrofuran (10 mL chromatographic grade, Tianjin Fuyu Fine Chemical Co., Ltd., China), then the mixture solution was filtered using a 0.2 μm membrane filter and degassed by sonication [12,40]. The flow rate was 1 ml min^-1^. Calibration curves were obtained based on several concentrations of a GABA standard (Sigma, USA), and the coefficient of determination (*r*^*2*^) was greater than 0.999.

### RNA isolation and preparation

The largest measured difference in concentration of GABA between the control group and experiment group was at 36 h of fermentation, and the GABA concentration of the experiment group was also highest at that time, so bacterial cells of 36 h were harvested by centrifugation (10000 × *g* for 10 min at 4°C) and the total RNA was extracted by RNAprep pure Cell/Bacteria Kit (Beijing Tiangen Biotech Co., Ltd., China) following the manufacturer’s instructions. The purity and concentration of RNA were measured by NanoDrop 2000 (Thermo Scientific, USA), and integrity was examined by electrophoresis using a 1% agarose gel. 3 μg RNA was used for RNA sample preparations and rRNA was removed using Ribo-Zero^TM^ rRNA Removal Kits (Illumina, USA). RNA libraries were generated using NEBNext^®^Ultra™ RNA Library Prep Kit for Illumina^®^ (NEB, USA) following manufacturer’s recommendations and index codes were added to attribute sequences of each sample. Briefly, mRNA was purified from total RNA via the MICROExpress kit (Ambion) according to the manufacturer’s instructions, and samples were resuspended in 15 μL RNase-free water [38,41].

### Library construction and sequencing

To generate whole transcriptome libraries, the resulting mRNA was cleaved to short fragments by adding NEB Next First Strand Synthesis Buffer (5X), then the first strand cDNA was synthesized by random hexamer primer and M-MuLV Reverse Transcriptase (RNase H^-^). Second strand cDNA was subsequently generated by adding buffer, dNTPs, DNA polymerase I and RNase H. After adenylation of 3’ ends of DNA, NEB Next sequencing adaptors with hairpin loop structure were ligated to the fragments. In order to select cDNA fragments of preferentially 150~200 bp in length, the library fragments were purified with AMPure XP system (Beckman Coulter, Beverly, USA), and were enriched by 15 cycles of linear PCR amplifications with Phusion High-Fidelity DNA polymerase, Universal PCR primers and Index (X) Primer. Finally, PCR products were purified (AMPure XP system) and library quality was assessed on the Agilent Bioanalyzer 2100 system. The library preparations were sequenced using the Illumina Hiseq^TM^ 2500 platform and paired-end reads were generated.

### Sequenced reads processing and mapping

After sequencing, the sequenced reads (raw reads) were initially assessed for sequencing error rate by Phred score, Q_phred_ [42]. Error rate distribution along reads of four sequenced sample is listed in the supplementary information. Then raw reads were processed through in-house perl scripts, and clean reads were selected from raw reads by removing adapter-related reads, ploy-N reads (unknown bases were more than 10%), and low-quality reads. Afterwards, all the downstream analyses were based on the resulting high-quality clean reads.

Although *L*. *plantarum* CGMCC 1.2437^**T**^ is the type strain of *L*. *plantarum* species, whole genome information of CGMCC 1.2437^**T**^ has not been completed, so a reference strain was chosen. Reference genome and gene model annotation files were downloaded from NCBI database (http://www.ncbi.nlm.nih.gov/, NCBI reference sequence: NC_004567.2, GenBank assembly accession: GCA_000203855.3, RefSeq assembly accession: GCF_000203855.3). Index of the reference genome was built using Bowtie v2.2.3 software and paired-end clean reads were aligned to the reference genome using TopHat v2.0.9 [43]. In the mapping, only mismatches less than 2 bases were allowed, and the multiple mapped genes to the reference was filtered. Subsequently, sequencing saturation analysis and correlation analysis (Pearson correlation) were performed to evaluate the quality of sequencing.

### Identification of differentially expressed genes

All libraries of clean reads were used to normalize the gene expression level using the DESeq method which provides statistical routines for determining digital gene differential expression by applying a model based on the negative binomial distribution [44]. In this study, read count instead of RPKM (Reads Per kb per Million reads) was used as a quantitative value to assess differential gene expression [45,46]. The absolute value of |log_2_ (readcount_MSG/ readcount_CTL)| and *P* value were manipulated to determine DEGs, and the log_2_ Ratio> 1 and *P*adj< 0.05 was used to judge the significance of gene expression discrepancy.

To further identify the functions and metabolic pathways relating to DEGs, Gene Ontology (GO) enrichment analysis and pathway enrichment analysis were carried out. All DEGs were mapped to GO terms in the database (http://www.geneontology.org/) and pathway terms in Kyoto Encyclopedia of Genes and Genomes (KEGG) database (http://www.genome.jp/kegg/). For GO enrichment, the method of GOseq was applied when performing GO analysis on RNA-seq data [47], and the statistical enrichment of DEGs in KEGG pathways was performed using the KOBAS software package [48].

### Relative expression analysis of genes involved in GABA-related pathways

To identify expression of genes of GABA-related pathways, as well as to verify RNA-seq results, real-time reverse transcriptome PCR (RT-qPCR) was performed. The RT-qPCR was performed as previously described [49], and each gene amplification included five replicates. All gene quantifications were performed with glyceraldehyde-3-phosphate dehydrogenase (GAPDH) as an internal standard, and the relative quantification of gene expression was analyzed by using the standard formula 2^-[(Et-Rt)-(Ec-Rc)]^. *CT* is the cycle number where the amplified target reaches the defined threshold; Et is the *CT* of the experimental gene in treated samples, Rt is the *CT* of GAPDH in treated samples, Ec is the *CT* of the experimental gene in control samples, and Rc is the *CT* of GAPDH in control samples [50]. Specific primers of GADPH and DEGs related to the GABA metabolic pathways were designed using Primer Premier 5.0 software.

### Statistics

Statistical analysis was performed comparing data of GABA fermented production between experimental and control groups. Data were expressed as the means ± standard deviations (SD) of triplicate determinations in two dependent experiments. To test the significance between different groups, one-way ANOVA, followed by the least-significant difference (LSD) were done by using SPSS 17.0 software (SPSS Inc., Chicago, IL, USA). *P* < 0.05 was used as the criterion of statistical significance.

## Results

### Effect of L-MSG on strain growth and GABA concentration

**Fig 1:**
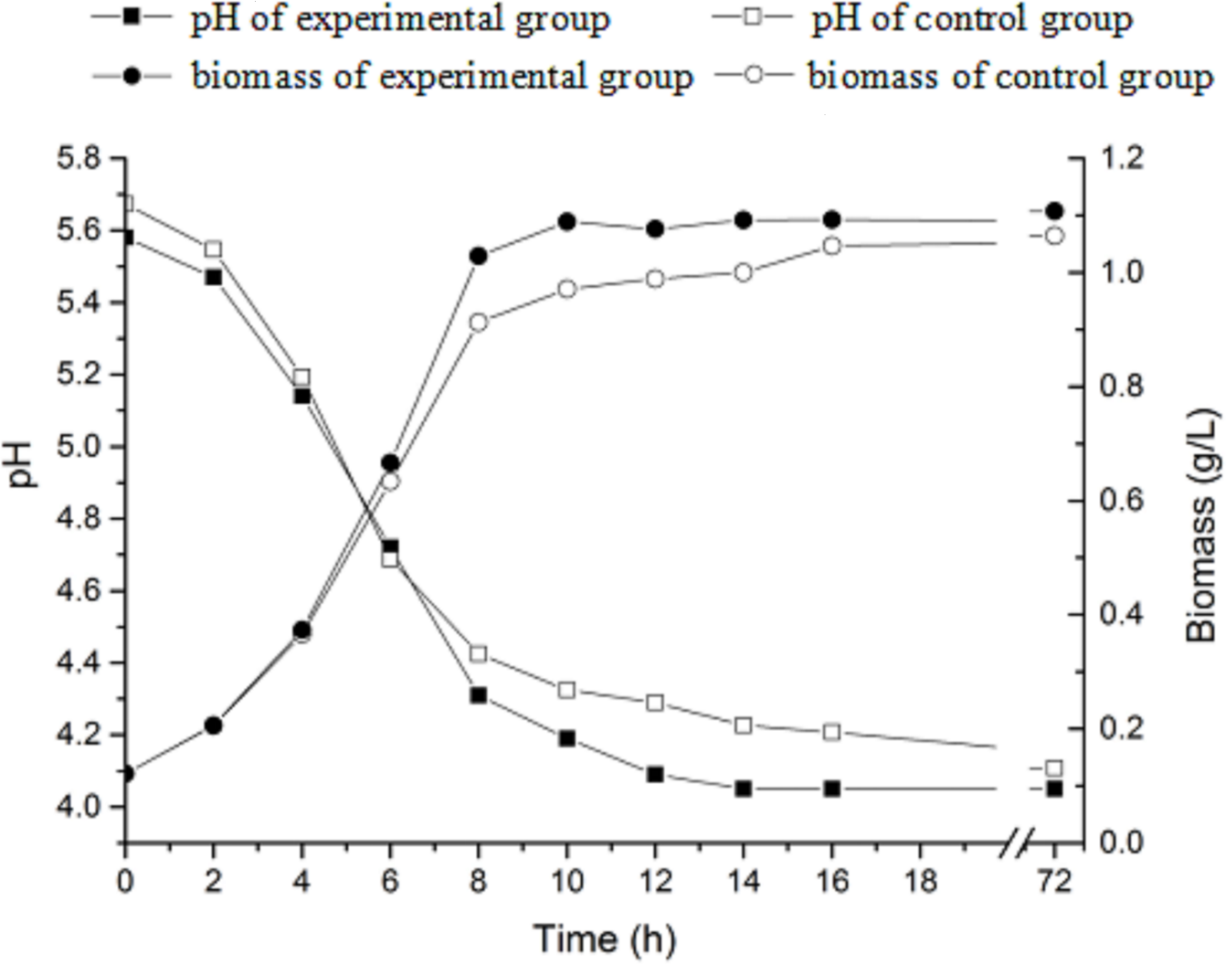
Comparison of pH and biomass in CDM culture with or without L-MSG. The solid square and circle show the pH and biomass of the experimental group. The hollow square and circle show the pH and biomass of the control group.

The pH values and biomass were not significantly affected by L-MSG addition. In both fermentation systems, strain growth stopped at 16 h and biomass was maintained at about 1.1 g L^-1^ during the rest time of the fermentation (Fig 1). The pH decreased from an initial highest value with the maximum rates of 0.22 ± 0.02 and 0.28 ± 0.02 h^-1^ to 4.05 ± 0.02 and 4.17± 0.02, then remained stable until the end of fermentation.

**Fig 2:**
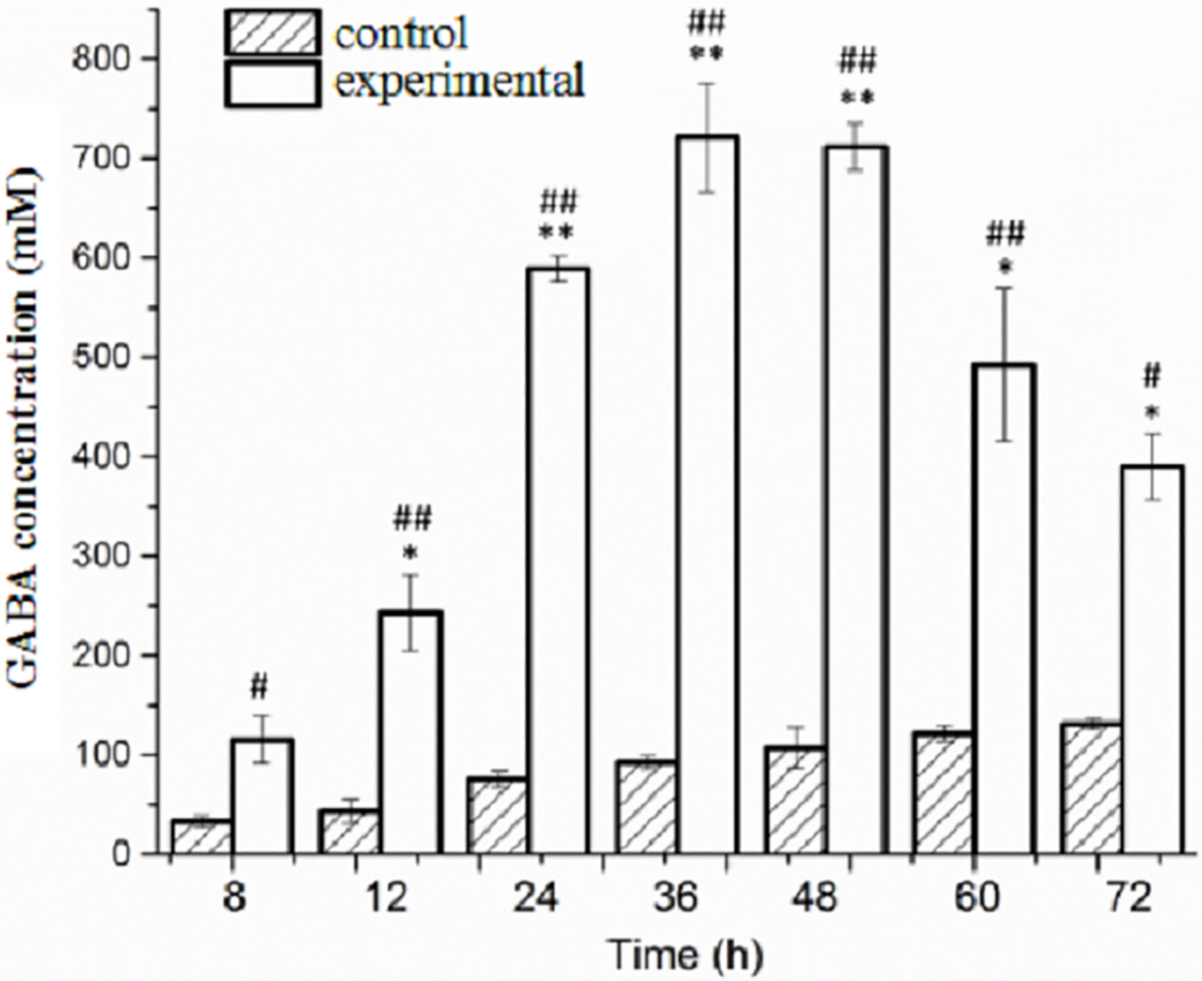
GABA concentrations at different time in CDM culture with or without L-MSG. ^#^, *P<*0.05; ^##^, *P<*0.01 indicated statistically significant differences between the glutamate group and control group at each time point.*, *P<*0.05; **, *P<*0.01 indicated statistically significant differences compared with the GABA concentration at 8 h of experimental group.

MSG, the substrate for GABA synthesis, significantly affected the concentration of GABA synthesized by the strain during fermentation (Fig 2). In the presence of L-MSG, GABA concentration accumulated rapidly for the experimental group during the first 36 hours of the fermentation and reached the peak value (721.35 mM) at 36 h, which was 7.77 fold greater than that of the control group. After that, the GABA concentration of experimental group decreased slowly to 390.49 mM at the end of fermentation, but it was still significantly higher than control group (*P* = 0.031). However, GABA was constantly synthesized at a relatively lower rate in the medium of the control group.

### Analysis of libraries and reads mapping

Illumina Hiseq^TM^ 2500 platform was used to perform high-throughput sequencing analysis of four *L*. *plantarum* CGMCC 1.2437^**T**^ libraries (LP_MSG1, LP_MSG2, LP_CTL1, LP_CTL2) to investigate the transcriptomic response to L-MSG induction during the fermentation. Nearly 8 million raw reads per library were generated and above 96.60% of total reads were identified as clean reads (S1 Fig) before mapping them to the reference genome. Base identification accuracy of four libraries was high, which were presented by Q20 above 95% and Q30 above 90% (Table 1). The error rate distribution along reads was showed in S2 Fig. For the Illumina Hiseq^TM^ 2500 platform, the maximum length of one fragment read is 125 bp, and a high error rate mainly distributes across the first 6 bases.

**Table 1:** Major characteristics of four libraries.

Item	Library			
	LP_MSG1	LP_MSG2	LP_CTL1	LP_CTL2
**raw reads**	7888615	8082150	8131745	7538528
**clean reads**	7670351	7838772	7855564	7331364
	97.23%	96.99%	96.60%	97.25%
**Error rate (%)**	0.03%	0.04%	0.03%	0.04%
**Q20 (%)**	95.56%	95.55%	95.51%	95.34%
**Q30 (%)**	91.18%	91.15%	91.11%	90.78%
**GC content (%)**	44.54%	44.55%	44.76%	44.62%

LP_MSG1 and LP_MSG2 were two replicates experimental group; LP_CTL1 and LP_CTL2 were two replicates of control group.

### Reference strain selection

Di-directional sequencing method was used for amplified 16S rDNA and IGS fragments of *L*. *plantarum* CGMCC 1.2437^**T**^, then BLAST analysis was carried out with *L*. *plantarum* CGMCC 1.2437^**T**^ and WCFS1, with the results showing high homology (100% and 99%). The total clean reads of the four libraries were mapped to a reference genome of *L*. *plantarum* WCFS1. Among these reads, over 97% clean reads were successfully mapped to the reference database and 3074 mapped genes per library were simultaneously obtained, which suggested that the reference was suitable. In addition, 95.09–96.28% of the clean reads were uniquely mapped to the reference genome, 47.51–48.11% were mapped to the sense strand and 47.58–48.17% were mapped to the anti-sense strand (Table 2).

**Table 2:** Mapping results between sequencing data and reference data.

Item		LP_MSG1	LP_MSG2	LP_CTL1	LP_CTL2
**total reads**	TN^a^	15340702	15677544	15711128	14662728
**total mapped reads**	TN	15025199	15357080	15289545	14264315
	TP^b^	97.94%	97.96%	97.32%	97.28%
**multiple mapped reads**	TN	265644	262860	294754	321552
	TP	1.73%	1.68%	1.88%	2.19%
**uniquely mapped reads**	TN	14759555	15094220	14994791	13942763
	TP	96.21%	96.28%	95.44%	95.09%
**reads mapped to ‘+’**^**c**^	TN	7375844	7542946	7492618	6966186
	TP	48.08%	48.11%	47.69%	47.51%
**reads mapped to ‘-’**^**d**^	TN	7383711	7551274	7502173	6976577
	TP	48.13%	48.17%	47.75%	47.58%

^a^ Total Number

^b^ Total Percentage

^c^ reads mapped to the sense strand

^d^ reads mapped to the anti-sense strand.

LP_MSG1 and LP_MSG2 were two replicates of experimental group; LP_CTL1 and LP_CTL2 were two replicates of control group.

### Analysis of gene expression

**Fig 3:**
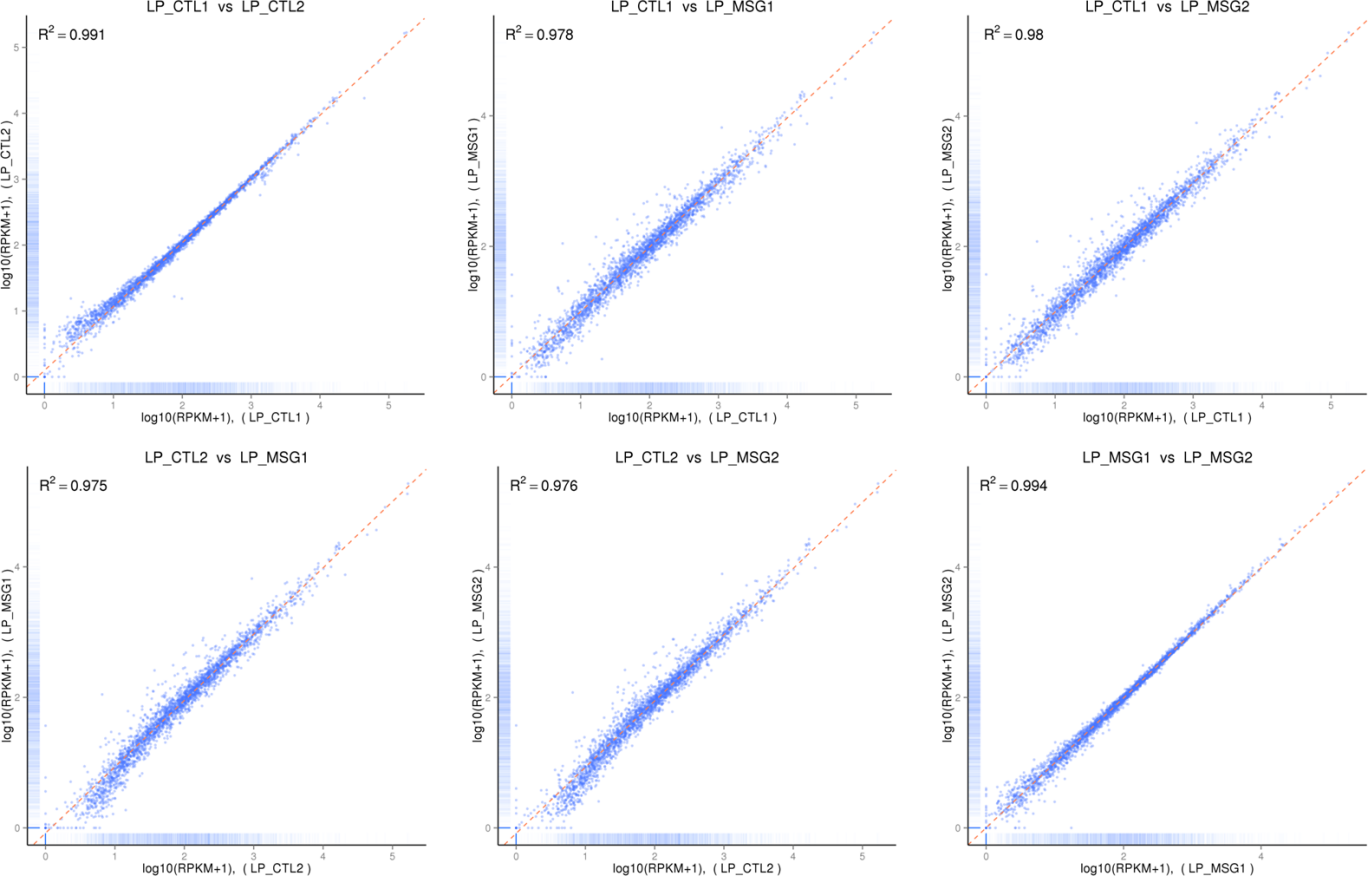
Correlation analysis between different samples. LP_MSG1 and LP_MSG2 were two replicates of experimental group; LP_CTL1 and LP_CTL2 were two replicates of control group.

The expression of genes may be directly reflected by their transcript abundances, with higher transcript abundance corresponding to higher gene expression. In RNA-seq, gene expression may be estimated by read counts mapped to the genome, and the relatively common practice is calculated using the RPKM (Reads Per kb per Million reads) method [51]. A total of 3074 genes were mapped, with nearly half of them expressed at a high level of RPKM (> 60), which suggested that these genes showed high expression across the four libraries. To estimate the accuracy of gene expression quantification, the saturation of expression level was tested (S3 Fig), and the accuracy of gene quantification increased with the increasing number of mapped reads and RPKM value. In the four libraries, almost all of genes could be quantified accurately when the percentage of mapped reads was above 90%, which suggested that the estimation of expression of uniquely mapped reads (above 95.09% of total raw reads) was reliable. In addition, similarities of expression profiles between two biological replicates of the same group and two different experimental groups were determined by Pearson correlation coefficient analysis (S4 Fig), with the log_2_-transcormed normalized read counts for the 3074 expressed genes were used as input. The two replicates in experimental and control groups showed high correlation with each other (R^2^ > 0.99); in contrast, the correlations between different treatments were 0.975, 0.976, 0.978 and 0.980, which were all lower than that of inter-group. Such results suggested that good consistency of gene expression profiles were presented between biological replicates, and different gene expression profiles were likely to appear because of L-MSG induction. The correlations were visualized using scatterplots (Fig 3). All the results suggested that our experiment designs and RNA-seq data were reliable.

### Analysis of differentially expressed genes

To identify genes in the *L*. *plantarum* CGMCC 1.2437^**T**^ that are differentially expressed in response to L-MSG induction, the numbers of read counts were compared. The genes differentially expressed between two transcriptomes were identified with the read counts, and the absolute value of log_2_ Ratio > 1 and *P*adj < 0.05 was used as the thresholds for significant differences in gene expression. The fold changes in expression (log_2_ readcount) ranged from -2.77 to +4.63. Among these DEGs, only 87 genes displayed significantly (*P*adj < 0.05) differential expression, 69 genes (one mapped to an unknown gene, possibly to the novel gene) were significantly upregulated and 18 genes (one gene mapped to small RNA) were significantly downregulated. Detailed information of the up-regulation and down-regulation significantly DEGs were shown in S1 Table.

### Identification of significantly DEGs induced by L-MSG

**Fig 4:**
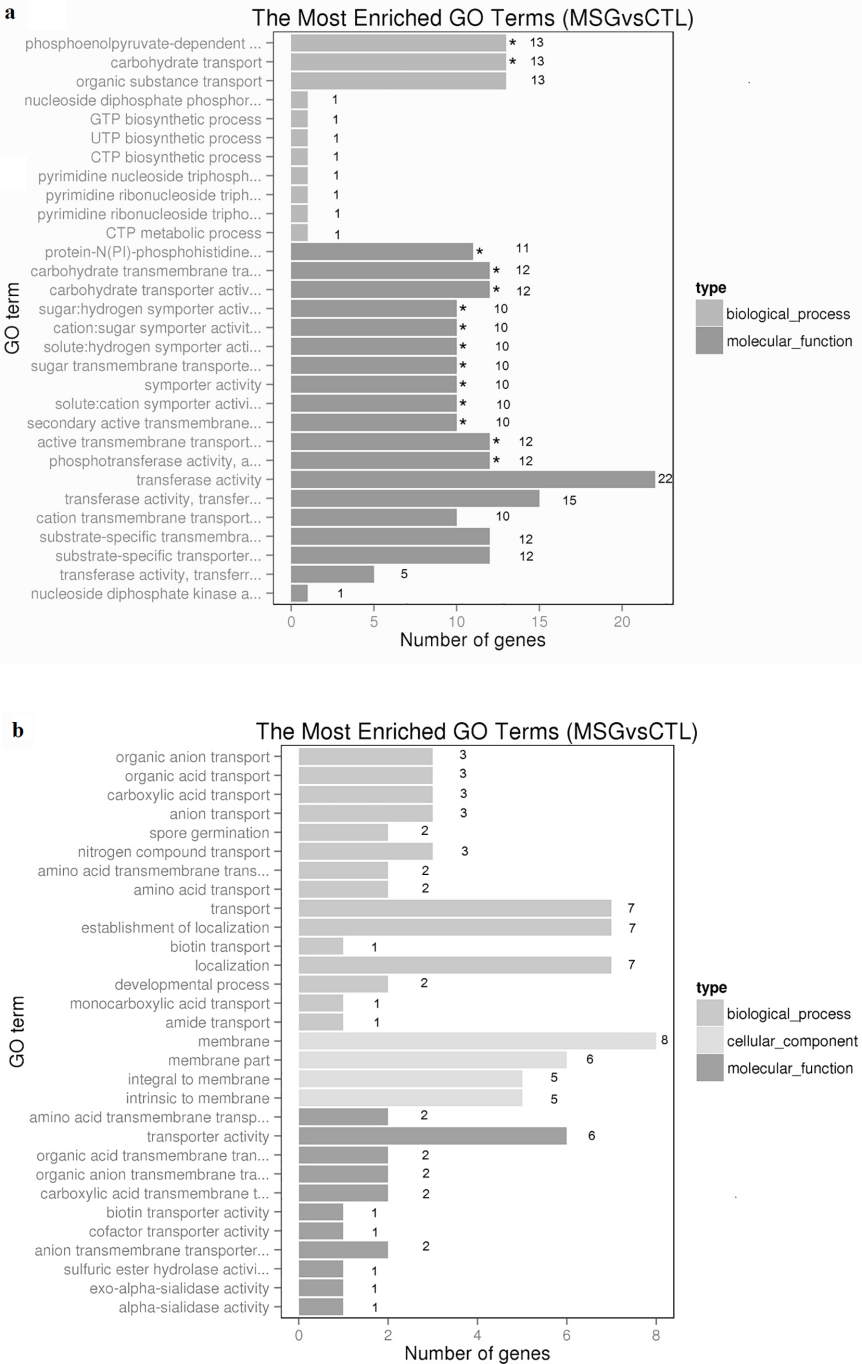
GO classification of significantly differentally expressed genes. Significantly up (a) and down (b) regulated DEGs between fermentation on CDM with L-MSG (MSG, experimental group) and without L-MSG (CTL, control group) were annotated into GO terms in three main GO categories.

In this study, the significantly DEGs induced by L-MSG were identified with GO and KEGG enrichment analysis. The GO classification assigned 67 significantly DEGs to 487 GO terms (S2 Table), and the most enriched 30 terms for up- and down- significantly DEGs were picked by default settings of analysis software respectively. Up-regulated genes were only involved biological process and molecular function (Fig 4A), while down-regulated genes covered three main GO categories: biological process, cellular process and molecular function [52] (Fig 4B).

**Fig 5:**
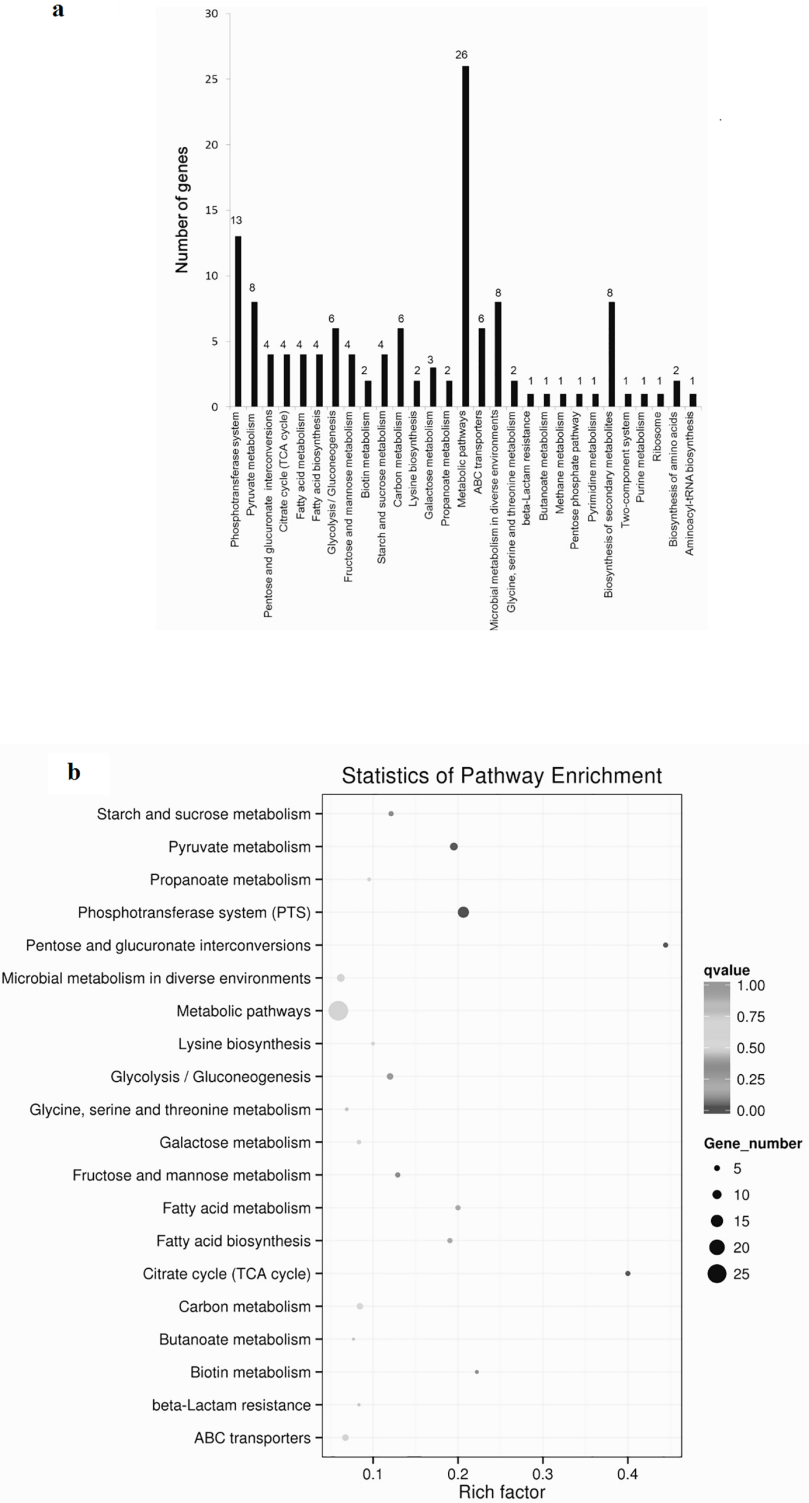
KEGG classifications of significantly differentially expressed genes. Significantly expressed genes between experimental group and control group were annotated into KEGG database and 29 relevant pathways were enriched (a). The 20 most enriched KEGG terms were screened (b).The color of the circle refers to the significance level of differential expressed genes enriched to the KEGG term and the size of circle dot means the number of differentially expressed genes.

In the KEGG annotation, 87 significantly DEGs were annotated into 29 metabolic pathways (Fig 5A and S3 Table). The most enriched 20 terms were picked by default settings of analysis software and the significant level of each term is shown in Fig 5B. Analysis data (corrected *p*-value) showed that the most significant metabolic pathways were the phosphotransferase system (PTS) (KEGG: lp102060), pyruvate metabolism (KEGG: lp100620), pentose and glucuronate interconversions (KEGG: lp10004), citrate cycle (KEGG: lp100020), fatty acid metabolism (KEGG: lp101212), fatty acid biosynthesis (KEGG: lp100061) and the glycolysis/gluconeogenesis pathway (KEGG: lp100010). Enrichment DEGs had the largest number in this entry of metabolic pathways, but the q value was not high, which suggests it was not a significant enrichment item.

## Discussions

This study examined induced synthesis of GABA based on L-MSG added to the culture medium as the only differential factor between experimental group and control group. In order to eliminate the influence of other irrelevant factors, a culture medium with known chemical components was used [30,31]. To further support the hypothesis that L-MSG was the only inducing factors, biomass and pH of culture broth in both experimental and control groups were not significantly different while GABA accumulation showed significant differences over time. This demonstrates that L-MSG could affect the metabolic pathways associated with GABA synthesis by the bacterium without inhibiting the growth of cells. Additionally, the differences in GABA concentration were most notable at 36–48 h of the fermentation process, when cell numbers were stable and the cellular growth was not observed. The differences relating to GABA concentration was primarily associated with different fermentation times and GABA-related metabolism. Smaller differences in GABA yield between the two groups was observed with the extended fermentation time, demonstrated by decreased levels among the experimental group and increased levels among the control group. Similar results could be seen in the study of Mazzoli et al [30], who speculated that although without addition of L-MSG, glutamine in the medium of control group could be converted into glutamic acid in the late fermentation, then utilized by GAD to generate small amount of GABA. And it can be hypothesized that GABA-transaminase effect led to the decomposition of GABA, which then caused the decrease of GABA in the experiment group [21].

In order to further understand the molecular mechanisms of GABA-induced synthesis, this study used high-throughput transcriptome sequencing of mRNA. Induction by L-MSG mediated notable changes at the transcriptional level were observed in metabolism related to PTS, pyruvate metabolism, and fatty acid synthesis. By comparing the metabolic pathways of the experimental and control groups in the key nodes corresponding to gene expression changes, the molecular mechanisms of GABA synthesis by L-MSG induced *L*. *plantarum* CGMCC 1.2437^T^ were inferred.

DEGs of L-MSG induced cells was mainly related to enrichment in PTS and carbohydrate metabolism. The PTS mainly relate to several phosphoenolpyruvate (PEP)-mediated sugar-phosphotransferase activities and carbohydrate metabolism involved in pyruvate metabolism and glycolysis. Various monosaccharides and disaccharides may be utilized by LAB, with three sugar transmembrane transport pathways for their cellular import. The first pathway involves active transport of glucose and the hydrolysis of ATP catalyzed by specific ATP enzymes [53], the second pathway is the symport pathway of glucose and hydrion or other solute compositions [54], and the third pathway is the process of phosphorylation through which glucose completed pyruvate metabolism together with getting into the cells [55]. In this study, significantly up-regulated genes in the experimental group were mainly associated with PEP-mediated PST pyruvate metabolism (pts4abc, pts5abc, pts7c, pts35c, pts35b). Results of the GO enrichment analysis identified determinants related to the PEP-dependent sugar-phosphotransferase system. Activation of this system stimulated transfer of phosphate groups of PEP, leading to production of pyruvic acid [55]. The accumulation of pyruvic acid stimulated its accelerated catabolism when it was induced by L-MSG. The study also showed that the genes encoding the pyruvate dehydrogenase complex (*pdhA*, *pdhB*, *pdhC* and *pdhD*), the gene encoding pyruvate oxidase (*poxL*), and the genes encoding pyruvate formate lyase (*pflA* and *pflB*) were up-regulated significantly. This means acetyl-CoA, acetyl phosphate and formate based produced by pyruvic acid were all activated by L-MSG [27,56]. In this case, pyruvic acid was broken down continuously, which coupled with no supplement of carbon source, may give rise to a rapid decrease of pyruvate accumulation. Clark et al. [57] found a pyruvate-dependent GABA-transaminase (GABA-T) exists in plants, and this enzyme, with pyruvate as an amino receptor induced GABA generation transamination of succinic semialdehyde. In the absence of pyruvate transamination activity of the GABA-T was greatly reduced, resulting in a large amount of GABA accumulation without transamination [58]. Transcriptome sequencing results of this study also showed that GABA-T (lp_1721, *gabT*) encoding gene expression in the experimental group was down-regulated by a factor of 0.26 times, indicating that the activity of GABA-T was inhibited.

Through the comprehensive analysis of GO enrichment and KEGG metabolic pathways, we hypothesize that the PEP-mediated sugar-phosphotransferase system was activated by L-MSG, then prompting accumulation of pyruvic acid produced by PEP, and this accumulation led to its catabolism. After 36 h of fermentation, the growth of cell reached mid-stationary period with no available carbon source due to the complete consumption of glucose in the medium, and together with its continuous decomposition led to a sharp decline of pyruvate. Therefore, GABA-T lost its transaminase activity in the absence of pyruvate leading to accumulation of GABA. After 48 h of fermentation, the accumulation of GABA in the experimental group began to decline: this maybe because the pyruvate metabolism pathways related to transamination of GABA changed, causing replenishment of intracellular pyruvate, and thus activity of GABA-T transaminase was restored. However, this hypothesis requires further study.

Results of this study showed that after induction by L-MSG, three enzymes (pyruvate decarboxylase complex-dihydrolipoic acid dehydrogenase (*pdhD*), pyruvate decarboxylase complex-dihydrolipoic acid transacetylase (*pdhC*), pyruvate decarboxylase complex alpha subunit (*pdhA*) and beta subunit (*pdhB*)) were up-regulated at the transcriptional level, which participated in the process of pyruvic acid converting to acetyl-CoA. This indicates that pyruvic acid was largely converted into acetyl-CoA. Meanwhile, the *accD* gene which encodes acetyl-CoA decarboxylase up-regulated 1.02 times, indicating acetyl-CoA continued to be used to produce malonyl-CoA by the catalysis of acetyl-CoA decarboxylase, which was further utilized in the synthesis of fatty acids. The increase in intracellular fatty acid synthesis can then improve the stability and toughness of cell membrane [59]. As described in previous studies, cells can enhance their survival ability in low pH, which is known as an “acid adaptive response”. As reported, the main mechanism of this phenomenon was the conversion of pyruvic acid to acetyl-CoA as indicated by up-regulation of pyruvate oxidase and phosphate acetyltransferase, and the formation of malonyl-CoA through up-regulation of acetyl-CoA decarboxylase. As a result fatty acid synthesis was promoted, and the stability of cells and resistance to acid improved [60–62]. In this study, the cells of the experimental group were kept in a high-salt environment from the beginning of fermentation, but the growth, reproduction and metabolism of the cells was unaffected. We hypothesize that L-MSG addition promoted the synthesis of fatty acids in cells, and so improved the hardness and density of the cell membrane, which enabled adaptation of the bacterial cells to the high-salt environment [63,64].

Metabolism of amino acids was also observed. Microbial GABA was mainly derived from glutamic acid and the decarboxylation reaction of glutamate, however, there was no clear explanation as to whether there were some connections between the synthesis or decomposition of GABA and the metabolism of glutamic acid or other amino acids. Transcriptome sequencing results of this study showed that no apparent changes of the expression of *gadB* (the encoding gene of GAD) were found between the experimental and control groups. Similar results were also described by Mazzoli et al [30]. They used glutamic acid as the substrate for *Lc*. *lactis* to induce the fermentation, and analyzed gene expression of induced cells. Results showed the expression of *gadB* in the glutamate-induced condition was similar to that of non-glutamate induced cells. These results indicated that biosynthesis of GAD has less dependence on glutamic acid or sodium glutamate in the substrates. In our research the synthesis of GABA in *L*. *plantarum* CGMCC 1.2437^T^ maybe regulated primarily by the catalytic activity of GAD, as the biosynthesis of GAD had little effect on the yield of GABA [30,65].

Pessione et al. [66] used proteomic approaches to analyse the metabolism of biogenic amines in two strains of *Lactobacillus*, and found that the biosynthesis of histidine decarboxylase and ornithine decarboxylase are dependent on their catalytic substrates. When histidine and ornithine existed, the biosynthetic capacity of these two decarboxylases was significantly higher than when no substrate was present. Further research reported that the biosynthesis of tryptophan decarboxylase in *Enterococcus faecalis* also had a dependence on tryptophan, which is its catalytic substrate [67]. Additional proteomic analysis may further clarify whether synthesis of GABA in *L*. *plantarum* CGMCC 1.2437^T^ is primarily regulated by the catalytic activity of GAD.

## Conclusion

The present study showed that L-MSG could induce *L*. *plantarum* CGMCC 1.2437^T^ GABA production. The potential molecular mechanism was predicted by functional gene and pathway enrichment analysis, suggesting: (1) L-MSG induced up-regulation of *gadB*, which facilitated GABA synthesis; (2) L-MSG induced down-regulation of *gabT*, which inhibited the degradation of GABA and contributed to GABA accumulation; (3) L-MSG induced conversion of glucose to succinic acid through glycolysis and pyruvate metabolism, and the succinic acid could also inhibit the catabolic pathways of GABA; (4) L-MSG could activate the synthesis of fatty acids, which increased the membrane permeability, contributed to substrata absorption, and facilitated to cell growth and metabolism. This research presents a theoretical foundation for GABA fermentation by *L*. *plantarum* at the molecular level, provides direction for further studies, and presents and approach which may be taken for related studies.

## Supporting information

S1 Fig Raw reads classification of RNA-seq of four samples. (DOCX)

S2 Fig Error rate of RNA-seq of four samples. (DOCX)

S3 Fig Saturation of genes expression level of four samples. (DOCX)

S4 Fig Pearson correlation coefficient analysis between different samples. (DOCX)

S1 Table Detail information of up-regulation and down-regulation differentially expressed genes. (XLSX)

S2 Table GO classification of differentially expressed genes. (XLSX)

S3 Table KEGG annotation of differentially expressed genes. (XLSX)
