# Supplementary material for: Transcriptomic response to GABA-producing Lactobacillus plantarum CGMCC 1.2437T induced by L-MSG
Source: PLoS One. 2018 Jun 12;13(6):e0199021. doi: 10.1371/journal.pone.0199021 (PMC5997328; doi:10.1371/journal.pone.0199021)
Supplement: S4 Fig — (DOCX) [file pone.0199021.s004.docx]

**
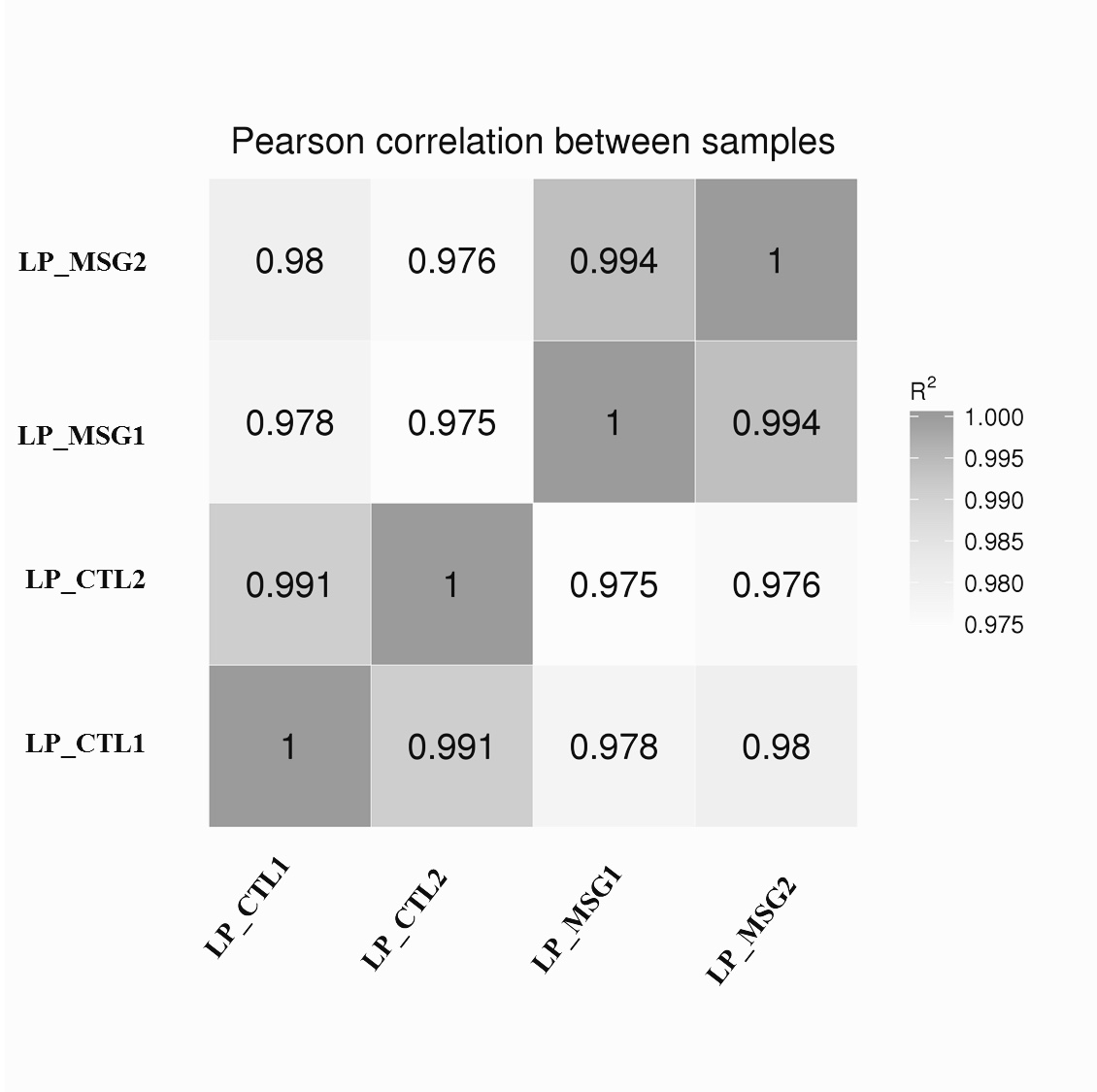
**

**S4 Fig. Pearson correlation coefficient analysis between different samples.**

LP_MSG1 and LP_MSG2 were two replicates experimental group;

LP_CTL1 and LP_CTL2 were two replicates of control group.
